# Loneliness and Social and Emotional Support Among Sexual and Gender Minority Caregivers

**DOI:** 10.1001/jamanetworkopen.2024.51931

**Published:** 2024-12-13

**Authors:** Zhigang Xie, Hanadi Hamadi, Kassie Terrell, Laggy George, Jennifer Wells, Jiaming Liang

**Affiliations:** 1 Department of Public Health, University of North Florida, Jacksonville; 2 Department of Health Administration, University of North Florida, Jacksonville; 3 School of Public Health, University of Texas Health Science Center at Houston, San Antonio

## Abstract

**Importance:**

Sexual and gender minority (SGM) adults in the US are more likely than their non-SGM counterparts to provide informal care to their family members and/or friends. Caregiving can impose substantial physical, mental, and social connection issues on caregivers.

**Objective:**

To examine the associations among loneliness, lack of social and emotional support, sexual orientation, gender identity, and informal caregiving status.

**Design, Setting, and Participants:**

This population-based, cross-sectional study used data from the 2022 Behavioral Risk Factor Surveillance System collected from noninstitutionalized US civilian residents aged 18 years or older. Data analysis was conducted from June to July 2024.

**Main Outcomes and Measures:**

The primary dependent variables included self-reported dichotomized (yes vs no) loneliness and lack of social and emotional support. The primary independent variables were sexual orientation (straight, gay or lesbian, bisexual, or something else), gender identity (cisgender and transgender), and informal caregiving status (yes vs no). A binomial distribution and log link function were used to derive adjusted prevalence ratios (APRs) and their respective 95% CIs for each outcome measure.

**Results:**

The analytic sample included 43 693 US adults (23 223 [51.6%] female at birth). In multivariable regression analyses adjusting for sociodemographic and health factors, among caregivers only, and compared with straight individuals, loneliness was significantly more likely among lesbian or gay individuals (APR, 1.30; 95% CI, 1.11-1.51), bisexual individuals (APR, 1.26; 95% CI, 1.12-1.43), and those who identify as something else (APR, 1.26; 95% CI, 1.09-1.46). Similarly, for noncaregivers, lesbian or gay individuals (APR, 1.34; 95% CI, 1.15-1.57), bisexual individuals (APR, 1.47; 95% CI, 1.34-1.61), and individuals identifying as something else (APR, 1.41; 95% CI, 1.25-1.58) experienced significantly higher risk of loneliness than straight noncaregivers. Transgender caregivers were more likely than cisgender caregivers to report loneliness (APR, 1.34; 95% CI, 1.24-1.46). In terms of lack of social and emotional support, bisexual caregivers (APR, 1.21; 95% CI, 1.00-1.48) and caregivers identifying as something else (APR, 1.34; 95% CI, 1.09-1.66) were significantly more likely than straight caregivers to report a lack of social and emotional support. Similarly, both bisexual noncaregivers (APR, 1.44; 95% CI, 1.28-1.63) and noncaregivers identifying as something else (APR, 1.38; 95% CI, 1.18-1.61) experienced a significantly higher risk of lacking support compared with straight noncaregivers.

**Conclusions and Relevance:**

In this cross-sectional study of social connections, SGM adults experienced significantly higher levels of loneliness compared with straight adults, irrespective of caregiving status. Furthermore, caregiving exacerbated these disparities across SGM subgroups. Patterns of lack of social and emotional support mirrored those of loneliness across different SGM and caregiving subpopulations, with nuances emerging upon adjustment for individual characteristics.

## Key Points

**Question:**

Is informal caregiving associated with perceived social connections (loneliness and lack of social and emotional support) differently among US adults with various sexual orientations and gender identities?

**Findings:**

In this population-based, cross-sectional study of 43 693 US adults, there was a consistently higher risk of loneliness among various sexual and gender minority groups, as well as caregivers, compared with their specific counterparts. In addition, individuals who identified as bisexual reported significantly greater lack of social and emotional support, regardless of their caregiving status.

**Meaning:**

These findings suggest that sexual and gender minority caregivers, particularly those who identify as bisexual, experience poorer social connections vs their straight counterparts.

## Teaser

This cross-sectional study examines the associations among loneliness, lack of social and emotional support, sexual and gender minority (SGM) status, and informal caregiving.

## Introduction

In the current landscape of US health care, informal unpaid caregiving provided by family members and friends is indispensable for managing diseases and ensuring long-term care in residential settings.^1^ As highlighted in the 2020 report by the American Association of Retired Persons and the National Alliance for Caregiving,^2^ more than one-fifth of US adults engaged in informal caregiving in 2020, and this number is projected to continue increasing. Notably, individuals who identify as sexual and gender minorities (SGM) are disproportionately represented among caregivers,^3,4,5^ despite often reporting strained relationships with their families of origin, whom they frequently support.^6,7,8,9,10^ Moreover, within the SGM umbrella, variations exist among subgroups in their caregiving roles; for instance, gay or lesbian adults demonstrate higher rates of caregiving compared with their bisexual counterparts.^3,4,11,12^

Although caregiving can foster familial bonds and a sense of fulfillment, its demands often curtail caregivers’ social engagements, limiting their ability to maintain relationships, participate in social activities, and pursue personal interests outside caregiving responsibilities.^9,13^ Withdrawal from social activities due to caregiving can intensify feelings of loneliness and diminish social support,^14,15,16,17^ which are critical factors shaping mental health,^18,19,20,21^ physical health,^20,22,23^ and overall quality of life.^24,25,26,27^ Given the already documented challenges in social connections and mental health within SGM communities,^13,28,29,30^ this issue can be potentially compounded among SGM caregivers, who may have fewer social resources to begin with.

Multiple systematic reviews^28,31,32,33^ have explored loneliness and the lack of social and emotional support among caregivers or SGM populations. These studies investigated these 2 populations separately but consistently demonstrated that both caregiving and SGM status are associated with increased loneliness and reduced support. Research focusing on caregiving within the SGM community has largely concentrated on direct health outcomes, such as the perceived mental and physical health of the caregiver.^22,27,30,34^ These findings indicate that caregivers generally face worse self-reported health outcomes compared with noncaregivers, with lesbian, gay, and bisexual caregivers being especially vulnerable.^3,34^ However, given the established connections between loneliness, lack of social and emotional support, and health outcomes, new research investigating the combined effects of SGM status and caregiving on loneliness and social support could provide actionable strategies to address health disparities, particularly for SGM adults who engage in informal caregiving.

It is important to note that sexual orientation and gender identity are distinct and nonbinary concepts; for instance, the umbrella term *SGM* encompasses various sexual and gender identities.^35,36^ Thus, to delve into these complexities, particularly across diverse SGM subpopulations, this study aimed to explain the associations among loneliness, lack of social and emotional support, sexual orientation (straight, gay or lesbian, bisexual, and something else), gender identity (cisgender and transgender), and informal caregiving status. By exploring these intersectional dynamics, we can better understand the nuanced impacts on social well-being within the SGM community, informing targeted interventions and support strategies.

## Methods

### Data Source

This cross-sectional study used secondary self-reported data from the 2022 Behavioral Risk Factor Surveillance System (BRFSS), a state-representative survey covering US states and participating US territories. BRFSS conducted state-based surveys via landline and cellular telephones among noninstitutionalized US civilian residents aged 18 years and older.^37^ The survey included a core section with standardized health-related questions that were mandatory for all participating states and territories to administer.^37^ In addition to the core section, an optional module that covered topics such as social determinants of health, sexual orientation, and gender identity, was implemented only in states that elect to collect such data.^37^ Individual states also had the flexibility to add specific questions to the BRFSS questionnaire. In 2022, 6 states (Georgia, Ohio, Oklahoma, Utah, Washington, and Wisconsin) included the sexual orientation and gender identity, caregiver, and social determinants and health equity modules. The validated survey instrument and detailed methods are available on the BRFSS website.^37^ Because the BRFSS data are publicly available and deidentified, our study did not require written informed consent for participant information, in accordance with 45 CFR §46. The institutional review board at the University of North Florida deemed this research exempt from review. Our study adheres to the Strengthening the Reporting of Observational Studies in Epidemiology (STROBE) reporting guidelines.^38^

### Study Sample

In this study, we restricted the analytic sample to individuals from 6 states who were asked about caregiving activities, SGM status, loneliness, and lack of social and emotional support (47 053 individuals). We further excluded those who answered “don’t know,” refused to answer, or were missing responses to questions related to caregiving, sexual orientation, gender identity, loneliness, and lack of social and emotional support (44 602 individuals). Finally, we also excluded those with missing information on selected sociodemographic covariates.

### Dependent Variables

This study included 2 primary dependent variables. The first variable, loneliness, was based on responses to the question, “How often do you feel socially isolated from others? Is it always, usually, sometimes, rarely, never, don’t know/not sure, refused?” Following the previous study,^13^ we dichotomized this variable with the responses “always/usually/sometimes” coded as “yes,” and “rarely/never” coded as “no.” Similarly, the second binary dependent variable, lack of social and emotional support, was created using responses to the question, “How often do you get the social and emotional support that you need? Is that always, usually, sometimes, rarely, never, don’t know/not sure, refused?” Lack of support (responses of “sometimes/rarely/never)” was coded as “yes,” and “always/usually” was coded as “no.”

### Independent Variables and Covariates

The primary independent variables of interest included (1) caregiving status (yes or no), (2) sexual orientation (straight, lesbian or gay, bisexual, and something else), and (3) gender modality (cisgender or transgender). Following the previous study,^13^ covariates included age (18-49 vs ≥50 years), sex at birth (male vs female), marital status (married or partnered vs not married or partnered), education attainment (less than college vs college or higher), annual household income (<$50 000, ≥$50 000, or unknown), employment status (employed vs unemployed), number of adults living in household (1 or ≥2), number of children living in household (0 or ≥1), depression history (yes vs no), and general health (excellent, very good, or good vs fair or poor). Self-reported race and ethnicity were collapsed to only 2 categories, non-Hispanic White and other (ie, American Indian or Alaskan Native, Asian, Black, Hispanic, Native Hawaiian or other Pacific Islander, and multiracial) because of insufficient cell sample size of caregivers from minoritized racial and ethnic groups. Race and ethnicity are included in this study because they potentially influence social connections among individuals with diverse sexual orientations and gender identities in unique ways. Other variables related to caregiving included the caregiver’s relationship to the recipient of care, the duration of caregiving, the average number of hours spent caregiving per week, the recipient’s primary condition, management of personal care, and management of household tasks. All the information was self-reported.

### Statistical Analysis

Following the complex sampling design of the BRFSS, we conducted weighted analyses using raking weights provided with the dataset.^39^ These weights adjust for respondent characteristics such as sociodemographic factors, telephone type (landline or cellular), and geographic location. Aligned with our study objectives, one set of analyses examined caregivers and noncaregivers by sexual orientation, while another set focused on caregivers and noncaregivers by gender identity.

We first compared differences in the baseline characteristics of the study sample based on sexual orientation and gender identity and caregiving status using Wald χ^2^ tests. Subsequently, weighted frequencies and 95% CIs were calculated on the basis of caregiving status and sexual orientation and gender identity. Using these weights, we used log-binomial regression models with the PROC GENMOD procedure in SAS software, using a binomial distribution and log link function to derive adjusted prevalence ratios (APRs) and their respective 95% CIs for each outcome measure. Statistical significance was defined as a 95% CI that did not contain the reference value of 1. These models were adjusted for significant sociodemographic factors presented in Table 1. Furthermore, we used similar analyses for subgroup analyses. All statistical analyses were performed using SAS version 9.4 (SAS Institute). Data analysis was conducted from June to July 2024.

**Table 1.** Participants’ Characteristics by Sexual Orientation or Gender Identity and Caregiving Status

Characteristics	Study sample, No. (weighted %) (N = 43 693; weighted N = 15 608 703)													
	Sexual orientation and caregiving status									Gender modality and caregiving status				
	Straight caregiver		Lesbian or gay caregiver		Bisexual caregiver		Something else caregiver		*P* value	Cisgender caregiver		Transgender caregiver		*P* value
	No	Yes	No	Yes	No	Yes	No	Yes		No	Yes	No	Yes	
Age, y														
18-49	11 369 (51.8)	2194 (41.5)	373 (69.8)	67 (49.9)	957 (89.3)	242 (85.5)	376 (77.1)	122 (81.0)	<.001	12 912 (54.3)	2573 (44.7)	163 (87.2)	52 (86.6)	<.001
≥50	20 597 (48.2)	5796 (58.5)	348 (30.2)	117 (50.1)	252 (10.7)	80 (14.5)	253 (22.9)	60 (19.0)		21 394 (45.7)	6034 (55.3)	56 (12.8)	19 (13.4)	
Sex at birth														
Male	15 874 (51.2)	3254 (42.1)	427 (58.9)	94 (54.5)	408 (32.0)	92 (26.4)	266 (39.2)	55 (28.7)	<.001	16 869 (50.3)	3471 (41.3)	106 (39.5)	24 (30.0)	<.001
Female	16 462 (48.8)	4825 (57.9)	299 (41.1)	92 (45.5)	807 (68.0)	234 (73.6)	374 (60.8)	130 (71.3)		17 827 (49.7)	5233 (58.7)	115 (60.5)	48 (70.0)	
Race and ethnicity														
Non-Hispanic White	26 344 (67.9)	6825 (72.7)	561 (61.8)	142 (64.8)	916 (64.6)	248 (69.4)	444 (59.0)	139 (63.5)	<.001	28 092 (67.4)	7308 (72.3)	173 (72.8)	46 (60.8)	<.001
Other^a^	5992 (32.1)	1254 (27.3)	165 (38.2)	44 (35.2)	299 (35.4)	78 (30.6)	196 (41.0)	46 (36.5)		6604 (32.6)	1396 (27.7)	48 (27.2)	26 (39.2)	
Marital status														
Married	19 608 (59.2)	5501 (63.1)	330 (42.6)	94 (48.6)	530 (38.6)	153 (42.5)	259 (35.0)	82 (39.7)	<.001	20 649 (57.5)	5796 (61.4)	78 (30.3)	34 (31.9)	<.001
Not married	12 728 (40.8)	2578 (36.9)	396 (57.3)	92 (51.4)	685 (61.4)	173 (57.5)	381 (65.0)	103 (60.3)		14 047 (42.5)	2908 (38.6)	143 (69.7)	38 (68.1)	
Education														
Less than college	8596 (34.7)	1911 (32.8)	163 (37.3)	33 (17.9)	349 (34.7)	85 (34.2)	239 (48.5)	59 (40.4)	<.001	9274 (35.0)	2063 (32.6)	73 (41.9)	25 (48.7)	.009
College or higher	23 740 (65.3)	6168 (67.2)	563 (62.7)	153 (82.1)	866 (65.3)	241 (65.8)	401 (51.5)	126 (59.6)		25 422 (65.0)	6641 (67.4)	148 (58.1)	47 (51.3)	
Annual household income, $														
<50 000	9031 (27.9)	2467 (34.2)	235 (35.6)	66 (31.6)	400 (33.6)	120 (39.3)	271 (40.4)	62 (37.7)	<.001	9857 (28.5)	2693 (34.6)	80 (40.3)	22 (28.4)	<.001
≥50 000	18 005 (54.9)	4427 (51.0)	399 (50.9)	101 (60.3)	607 (45.2)	155 (46.5)	221 (29.7)	79 (40.3)		19 149 (54.0)	4731 (50.8)	83 (28.9)	31 (39.3)	
Unknown	5300 (17.2)	1185 (14.9)	92 (13.5)	19 (8.1)	208 (21.2)	51 (14.3)	148 (29.8)	44 (22.0)		5690 (17.5)	1280 (14.7)	58 (30.8)	19 (32.3)	
Employment														
Employed	16 374 (59.6)	3751 (51.7)	442 (66.1)	105 (67.0)	775 (65.2)	203 (64.8)	328 (54.2)	92 (48.0)	<.001	17 803 (59.9)	4109 (52.5)	116 (53.5)	42 (61.4)	<.001
Unemployed	15 962 (40.4)	4328 (48.3)	284 (33.9)	81 (33.0)	440 (34.8)	123 (35.2)	312 (45.8)	93 (52.0)		16 893 (40.1)	4595 (47.5)	105 (46.5)	30 (38.6)	
No. of adults living in household														
1	13 218 (32.3)	2958 (32.9)	291 (32.7)	71 (36.0)	361 (23.3)	90 (25.1)	256 (30.3)	58 (23.6)	<.001	14 061 (32.0)	3162 (32.5)	65 (19.4)	15 (12.9)	<.001
≥2	19 118 (67.7)	5121 (67.1)	435 (67.3)	115 (64.0)	854 (76.7)	236 (74.9)	384 (69.7)	127 (76.4)		20 635 (68.0)	5542 (67.5)	156 (80.6)	57 (87.1)	
No. of children living in household														
0	23 829 (65.3)	6162 (69.1)	624 (84.3)	163 (83.3)	844 (65.2)	198 (57.3)	483 (71.4)	134 (70.2)	<.001	25 609 (65.8)	6603 (68.9)	171 (76.8)	54 (66.1)	0.001
≥1	8507 (34.7)	1917 (30.9)	102 (15.7)	23 (16.7)	371 (34.8)	128 (42.7)	157 (28.6)	51 (29.8)		9087 (34.2)	2101 (31.1)	50 (23.2)	18 (33.9)	
Depression history														
Yes	6435 (19.1)	2226 (28.5)	265 (37.5)	93 (53.7)	671 (58.2)	207 (63.0)	258 (40.8)	100 (55.3)	<.001	7495 (21.5)	2582 (31.3)	134 (64.0)	44 (63.4)	<.001
No	25 901 (80.9)	5853 (71.5)	461 (62.5)	93 (46.3)	544 (41.8)	119 (37.0)	382 (59.2)	85 (44.7)		27 201 (78.5)	6122 (68.7)	87 (36.0)	28 (36.6)	
General health														
Good-excellent	27 305 (85.2)	6642 (81.0)	608 (84.1)	154 (88.5)	997 (81.1)	243 (75.8)	450 (72.0)	133 (66.7)	<.001	29 199 (84.8)	7122 (80.6)	161 (75.1)	50 (74.5)	<.001
Poor-fair	5031 (14.8)	1437 (19.0)	118 (15.9)	32 (11.5)	218 (18.9)	83 (24.2)	190 (28.0)	52 (33.3)		5497 (15.2)	1582 (19.4)	330 (24.9)	22 (25.5)	

^a^ The other category included American Indian or Alaskan Native, Asian, Black, Hispanic, Native Hawaiian or other Pacific Islander, and multiracial. Race and ethnicity were categorized into 2 levels to account for insufficient cell sample size of minoritized caregivers.

## Results

Of the 43 693 adults surveyed (23 223 [51.6%] female at birth; weighted number, 15 608 703 adults), 9.5% (95% CI, 9.1%-10.0%) identified as sexual minorities. Among these, 19.7% (95% CI, 19.1%-20.4%) of heterosexual individuals, 17.9% (95% CI, 14.1%-21.6%) of gay or lesbian individuals, 21.7% (95% CI, 18.5%-24.9%) of bisexual individuals, and 24.0% (95% CI, 19.7%-28.3%) of those identifying as something else reported having engaged in caregiving in the past month. In addition, approximately 1% identified as transgender. Among the transgender participants, 25.1% (95% CI, 18.3%-31.9%) reported caregiving experience, compared with 19.8% (95% CI, 18.2%-20.5%) of cisgender participants. Table 1 shows the distribution of participants’ sociodemographic characteristics categorized by sexual orientation, gender identity, and caregiving status. Within each different sexual orientation or gender identity subgroup, caregivers were more likely to be female, married, have higher education, and report depression history compared with noncaregivers.

### Loneliness

Table 2 presents the prevalence of loneliness and lack of social and emotional support by SGM and caregiving status. Straight noncaregivers reported the lowest prevalence of loneliness at 29.2% (95% CI, 28.3%-30.1%), followed by straight caregivers (39.2%; 95% CI, 37.3%-41.1%), lesbian or gay noncaregivers (46.4%; 95% CI, 40.1%-52.6%), lesbian or gay caregivers (52.6%; 95% CI, 41.3%-63.8%), bisexual noncaregivers (54.5%; 95% CI, 50.1%-58.8%), and bisexual caregivers (62.3%; 95% CI, 54.7%-70.0%). Caregivers identifying their sexual orientation as something else had the highest prevalence of loneliness at 64.3% (95% CI, 54.8%-73.8%). Likewise, the prevalence of loneliness varied by gender identity and caregiving status, ranging from 30.9% (95% CI, 30.1%-31.8%) among cisgender noncaregivers to 74.9% (95% CI, 62.2%-87.7%) among transgender caregivers. eTable 1 in Supplement 1 presents further detailed information on loneliness across various SGM groups.

**Table 2.** Prevalence of Loneliness and Lack of Social and Emotional Support by Sexual Orientation or Gender Identity and Caregiver Status

Variable	Prevalence, % (95% CI)	
	Loneliness	Lack of social and emotional support
Sexual orientation and caregiving status		
Straight		
Noncaregiver	29.2 (28.3-30.1)	22.3 (21.5-23.1)
Caregiver	39.2 (37.3-41.1)	32.8 (30.9-34.7)
Lesbian or gay		
Noncaregiver	46.4 (40.1-52.6)	29.3 (23.2-35.4)
Caregiver	52.6 (41.3-63.8)	26.2 (18.0-34.4)
Bisexual		
Noncaregiver	54.5 (50.1-58.8)	36.8 (32.4-41.1)
Caregiver	62.3 (54.7-70.0)	45.5 (37.2-53.7)
Something else		
Noncaregiver	51.5 (45.5-57.6)	39.1 (33.3-44.8)
Caregiver	64.3 (54.8-73.8)	53.6 (43.5-63.6)
Gender modality and caregiving status		
Cisgender		
Noncaregiver	30.9 (30.1-31.8)	23.4 (22.6-24.2)
Caregiver	41.1 (39.2-42.9)	33.7 (31.9-35.5)
Transgender		
Noncaregiver	70.3 (61.7-79.0)	37.6 (28.7-46.5)
Caregiver	74.9 (62.2-87.7)	50.3 (34.8-65.8)

In multivariable regression analyses (Table 3), among caregivers only, compared with straight individuals, loneliness was more likely among lesbian or gay individuals (APR, 1.30; 95% CI, 1.11-1.51), bisexual individuals (APR, 1.26; 95% CI, 1.12-1.43), and individuals who identify as something else (APR, 1.26; 95% CI, 1.09-1.46). Similarly, for noncaregivers, lesbian or gay individuals (APR, 1.34; 95% CI, 1.15-1.57), bisexual individuals (APR, 1.47; 95% CI, 1.34-1.61), and individuals identifying as something else (APR, 1.41; 95% CI, 1.25-1.58) experienced significantly higher risk of loneliness compared with straight noncaregivers. In addition, the APR (caregiver vs noncaregiver) ranged from 1.17 (95% CI, 1.02-1.35) for bisexual individuals to 1.37 (95% CI, 1.29-1.45) for straight individuals across the 4 sexual orientation groups.

**Table 3.** Risk of Loneliness and Lack of Social and Emotional Support in Regression Analyses

Variable	APR (95% CI)^a^	
	Loneliness	Lack of social and emotional support
Sexual orientation and caregiving status		
Caregiver		
Straight	1 [Reference]	1 [Reference]
Lesbian or gay	1.30 (1.11-1.51)	0.79 (0.58-1.09)
Bisexual	1.26 (1.12-1.43)	1.21 (1.00-1.48)
Something else	1.26 (1.09-1.46)	1.34 (1.09-1.66)
Noncaregiver		
Straight	1 [Reference]	1 [Reference]
Lesbian or gay	1.34 (1.15-1.57)	1.11 (0.89-1.39)
Bisexual	1.47 (1.34-1.61)	1.44 (1.28-1.63)
Something else	1.41 (1.25-1.58)	1.38 (1.18-1.61)
Straight		
Noncaregiver	1 [Reference]	1 [Reference]
Caregiver	1.37 (1.29-1.45)	1.44 (1.34-1.55)
Lesbian or gay		
Noncaregiver	1 [Reference]	1 [Reference]
Caregiver	1.32 (1.07-1.63)	1.03 (0.70-1.51)
Bisexual		
Noncaregiver	1 [Reference]	1 [Reference]
Caregiver	1.17 (1.02-1.35)	1.21 (0.98-1.50)
Something else		
Noncaregiver	1 [Reference]	1 [Reference]
Caregiver	1.22 (1.03-1.46)	1.40 (1.10-1.80)
Gender modality and caregiving status		
Caregiver		
Cisgender	1 [Reference]	1 [Reference]
Transgender	1.34 (1.24-1.46)	1.05 (0.63-1.76)
Noncaregiver		
Cisgender	1 [Reference]	1 [Reference]
Transgender	1.70 (1.58-1.84)	1.34 (1.06-1.68)
Cisgender		
Noncaregiver	1 [Reference]	1 [Reference]
Caregiver	1.35 (1.28-1.43)	1.42 (1.32-1.52)
Transgender		
Noncaregiver	1 [Reference]	1 [Reference]
Caregiver	1.07 (0.98-1.16)	1.11 (0.64-1.95)

Abbreviation: APR, adjusted prevalence ratio.

^a^ Adjusted for age, sex at birth, race and ethnicity, marital status, education, income, employment status, number of adults in the household, number of children in the household, depression history, and general health.

Comparable patterns emerged regarding gender identity and caregiving status. Among caregivers, transgender individuals were 1.34 times more likely to report loneliness than cisgender individuals (APR, 1.34; 95% CI, 1.24-1.46). This risk was also significantly higher among transgender noncaregivers, with an APR of 1.70 (95% CI, 1.58-1.84) compared with cisgender noncaregivers. The APRs for caregivers vs noncaregivers were 1.07 (95% CI, 0.98-1.16) in the transgender population and 1.35 (95% CI, 1.28-1.43) in the cisgender population.

### Lack of Social and Emotional Support

A consistently higher prevalence of lack of social and emotional support was observed among SGM noncaregivers and caregivers compared with straight or cisgender noncaregivers (Table 2). This prevalence ranged from a low of 22.3% (95% CI, 21.5%-23.1%) among straight noncaregivers to a high of 53.6% (95% CI, 43.5%-63.6%) among caregivers who identified as something else. Among different gender identities and caregiving statuses, the prevalence ranged from 23.4% (95% CI, 22.6%-24.2%) among cisgender noncaregivers to 50.3% (95% CI, 34.8%-65.8%) among transgender caregivers. eTable 2 in Supplement 1 shows further detailed information on the lack of social and emotional support across different SGM groups.

After adjusting for sociodemographic characteristics (Table 3), among caregivers, bisexual individuals (APR, 1.21; 95% CI, 1.00-1.48) and those identifying as something else (APR, 1.34; 95% CI, 1.09-1.66) were significantly more likely to report a lack of social and emotional support than straight caregivers. Similarly, among noncaregivers, bisexual individuals (APR, 1.44; 95% CI, 1.28-1.63), those who identified as something else (APR, 1.38; 95% CI, 1.18-1.61), and transgender individuals (APR, 1.34; 95% CI, 1.06-1.68) experienced a significantly higher risk of lacking support compared with straight noncaregivers. Furthermore, caregiving status was significantly associated with lacking support only in the straight population (APR, 1.44; 95% CI, 1.34-1.55), those whose sexual orientation was something else (APR, 1.40; 95% CI, 1.10-1.80), and the cisgender population (APR, 1.42; 95% CI, 1.32-1.52). No significant differences were observed between transgender and cisgender populations in the caregiver groups.

### Subgroup Analyses

To provide further insights, Table 4 shows the comparisons of loneliness and lacking social and emotional support between SGM caregivers and straight, cisgender noncaregivers. eTable 3 in Supplement 1 presents caregiving-related characteristics by sexual orientation and gender identity among caregivers. No statistically significant differences were observed across sexual orientation and gender identity groups. Comparable findings were observed after adjusting for sociodemographic and caregiving-related characteristics (eTable 4 in Supplement 1).

**Table 4.** Comparing the Risk of Loneliness and Lack of Social and Emotional Support Between Sexual and Gender Minority Caregivers and Straight or Cisgender Noncaregivers

Variable	APR (95% CI)^a^	
	Loneliness	Lack of social and emotional support
Sexual orientation and caregiving status		
Straight noncaregiver	1 [Reference]	1 [Reference]
Lesbian or gay caregiver	1.77 (1.53-2.06)	1.14 (0.83-1.57)
Bisexual caregiver	1.73 (1.54-1.95)	1.75 (1.45-2.11)
Something else caregiver	1.72 (1.49-1.98)	1.93 (1.58-2.37)
Gender modality and caregiving status		
Cisgender noncaregiver	1 [Reference]	1 [Reference]
Transgender caregiver	1.82 (1.69-1.95)	1.49 (0.89-2.49)

Abbreviation: APR, adjusted prevalence ratio.

^a^ Adjusted for age, sex at birth, race and ethnicity, marital status, education, income, employment status, number of adults in the household, number of children in the household, depression history, and general health.

## Discussion

We conducted this secondary cross-sectional analysis to explore the combined associations of SGM status and caregiving with loneliness and perceived social support among adults in the US. This study identified that SGM adults were more likely to take on informal caregiving roles and report higher levels of loneliness compared with their non-SGM counterparts. In addition, patterns of lacking social and emotional support were notably present among bisexual individuals and those who identified their sexual orientation as something else. There was a significantly higher risk of loneliness among caregivers across all SGM subpopulations, while the association between caregiving status and lacking social and emotional support was observed only in straight and cisgender populations. Our study supports previous findings but also highlights the varying outcomes across different SGM subpopulations, indicating the need for tailored interventions to address the unique challenges faced by each subgroup.

The higher risk of loneliness among SGM populations can be related to various family structure–related factors: SGM individuals are less likely to be partnered or married, less likely to have children, and more likely to live alone and be estranged from their families of origin than their non-SGM peers.^6^ For SGM caregivers, this risk may be even greater due to the added layers of caregiving responsibilities (ie, physical assistance and medication management)^19,23^ and related financial strains, marginalization, and unique challenges related to their sexual orientation and gender identity.^14,26,40,41,42^

Also, the discrimination against SGM individuals can force them to hide aspects of their identity, leading to mental exhaustion^9,43,44^ as they balance providing caregiving duties within a space that may be perceived as unsafe due to family members, friends, or health care practitioners who may be unsupportive of their sexual or gender identity.^17,45,46,47,48,49^ For example, SGM people who care for parents or loved ones who previously did not support or accept them often experience complex emotional challenges, including feelings of resentment, anger, and unresolved trauma.^8,47,50^

In addition, providing care can exacerbate feelings of isolation and conflict, as they navigate a relationship fraught with past pain while trying to provide care.^51^ This internal struggle can result in heightened stress and internal conflict.^9,42^ Although SGM caregivers may lean on chosen families and social support networks,^6,10,47^ caregiving responsibilities can disrupt these connections and limit their social interactions outside of home and work.^15,52,53^ Furthermore, internalizing negative societal attitudes can lead to feelings of shame, guilt, and low self-esteem, which can worsen their sense of loneliness.^16,43^

The insignificant difference in lacking social and emotional support between lesbian or gay and straight adults may be attributed to the increasing societal acceptance of lesbian and gay identities in recent years.^38,54,55^ There has been a surge in the number of support programs for SGM populations designed to offer support and community. These initiatives led to stronger community support networks for gay and lesbian individuals, which can help offset potential gaps in family support.^56^ Enhanced visibility^57,58^ and representation may also facilitate better access to resources for lesbian and gay individuals, particularly in health care settings.^54,55,59^ For instance, these resources in health care settings include the Gay and Lesbian Medical Association.^59^ Furthermore, lesbian and gay individuals may benefit more from the support from chosen families due to having a relatively larger and more supportive network,^3,5^ as they are more likely to come out to the people in their lives compared with other SGM subgroups.

In contrast, individuals who identify as bisexual or with other nonheteronormative identities may face distinct challenges in securing social support due to lower societal acceptance and visibility. They often encounter double discrimination, facing prejudice from both heterosexual and SGM communities,^11,13,22,60^ which can result in smaller support networks and heightened feelings of isolation. Furthermore, those who identify as something else may have more complex identities that are less understood, making it difficult to find supportive communities or individuals who relate to their experiences.

To address the deeply interconnected loneliness and lack of social and emotional support issues among SGM caregivers, it is crucial to create a more supportive and inclusive environment for SGM caregivers, addressing their unique needs and ensuring they have access to the resources and support they need.^60,61^ In addition to increased visibility of the aforementioned resources, it is crucial to update traditional caregiver policies and programs to include SGM caregivers.^10,49,55,56,62^ Changes at the policy level will enhance access to services and support currently available to non-SGM caregivers. In addition, advocacy for the inclusion of SGM individuals and caregivers in decision-making bodies and policy advisory groups is essential to ensure their perspectives are represented and discrimination is minimized. Policies should also recognize and support diverse family structures, including chosen families, which are especially important for SGM caregivers who may lack biological family support.^6,10^

At the community level, implementing cultural competency training for health care practitioners and social workers is necessary for practitioners to better understand SGM issues and the unique challenges faced by SGM caregivers.^34^ This includes expanding the definition and understanding of diversity, equity, and inclusion beyond the narrow definition of its reputation to honor and include all components of intersectionality. It is also important to support the development and maintenance of community centers that offer safe and supportive spaces for SGM caregivers to connect and access resources. These organizations facilitate the creation of social support networks and provide peer support groups specifically tailored to SGM caregivers.

At the individual level, it is vital that we increase access to mental health resources for SGM caregivers, including counseling and support groups that address both caregiving and SGM-specific issues. This includes providing inclusive and affirming care, as well as individualized coping strategies for clients^63,64^ with diverse sexual and gender identities (eg, avoiding heteronormative assumptions and biases, being aware of gender-affirming care options, and creating a safe and welcoming environment for all clients regardless of their SGM identity). Considering the diverse SGM subpopulations and their varying risks of loneliness and lack of support, it is essential to balance overarching strategies for the entire SGM community with targeted solutions that address the specific needs of individual subpopulations.

### Limitations

Although this study contributes to the limited research in this area, there are a few limitations. First, our sample was drawn from only several states that included both sexual orientation and gender identity and caregiving-related questions; as such, it should not be considered nationally representative because of state-to-state variations.^65^ Future research with a nationally representative sample would be beneficial. Second, to ensure sufficient observations in each category, we dichotomized the 2 dependent variables, which may lead to loss of nuances in the analysis. However, the additional analysis using the original classifications showed similar conclusions, and it suggested the dichotomized version dependent variables captured the main information. In addition, we also collapsed some covariates (eg, age and race and ethnicity) into 2 categories owing to the small sample size of SGM caregivers, and this approach may obscure important nuances across subpopulations. Although the large overall sample size from the BRFSS facilitated this analysis, it underscores the need to oversample minority populations in future national surveys. Third, the self-reported data may introduce recall and social desirability biases, and future studies using objective measures will be needed. In addition, the 2022 BRFSS asked participants whether they felt socially isolated, rather than measuring loneliness directly, which could lead to misunderstandings. This question will be revised in the 2023 BRFSS to address this issue more accurately; future researchers are encouraged to explore these intersections with the new question.

## Conclusions

In this cross-sectional study of social connections, SGM adults experienced significantly higher levels of loneliness compared with straight adults, irrespective of caregiving status. Furthermore, caregiving exacerbated these disparities across SGM subgroups. Patterns of lack of social and emotional support mirrored those of loneliness across different SGM and caregiving subpopulations, with nuances emerging upon adjustment for individual characteristics. Future studies using causal effect designs with increased patient and public involvement are essential for further exploring these interconnected factors.

## Notes

Supplement 1. **eTable 1. **Prevalence of loneliness by sexual orientation/gender identity and caregiver status **eTable 2.** Prevalence of social and emotional support by sexual orientation/gender identity and caregiver status **eTable 3.** Participants’ caregiving-related characteristics by sexual orientation/gender identity among caregivers (n=7,765, Weighted n=2,719,664) **eTable 4.** Risk of loneliness and lack of social and emotional support in regression analyses among caregivers

Supplement 2.
Data Sharing Statement
