# Supplementary material for: Loneliness and Social and Emotional Support Among Sexual and Gender Minority Caregivers
Source: JAMA Netw Open. 2024 Dec 13;7(12):e2451931. doi: 10.1001/jamanetworkopen.2024.51931 (PMC11645647; doi:10.1001/jamanetworkopen.2024.51931)
Supplement: Supplement 1. — eTable 1. Prevalence of loneliness by sexual orientation/gender identity and caregiver status eTable 2. Prevalence of social and emotional support by sexual orientation/gender identity and caregiver status eTable 3. Participants’ caregiving-related characteristics by sexual orientation/gender identity among caregivers (n=7,765, Weighted n=2,719,664) eTable 4. Risk of loneliness and lack of social and emotional support in regression analyses among caregivers [file jamanetwopen-e2451931-s001.pdf]

Supplemental Online Content

Xie Z, Hamadi H, Terrell K, George L, Wells J, Liang J. Loneliness and social and emotional support among sexual and gender minority caregivers. *JAMA Netw Open*. 2024;7(12):e2451931. doi:10.1001/jamanetworkopen.2024.51931

eTable 1. Prevalence of loneliness by sexual orientation/gender identity and caregiver status

eTable 2. Prevalence of social and emotional support by sexual orientation/gender identity and caregiver status

eTable 3. Participants' caregiving-related characteristics by sexual orientation/gender identity among caregivers (n=7,765, Weighted n=2,719,664)

eTable 4. Risk of loneliness and lack of social and emotional support in regression analyses among caregivers

This supplemental material has been provided by the authors to give readers additional information about their work.

© 2024 Xie Z et al. *JAMA Network Open*.

eTable 1. Prevalence of loneliness by sexual orientation/gender identity and caregiver status
(Question: How often do you feel socially isolated from others? Is it always, usually, sometimes, rarely, or never?)

	**Prevalence % (95%CI)**					***P***
	Always	Usually	Sometimes	Rarely	Never	
**Sexual Orientation and Caregiving Status**						<.001
Straight non-caregiver	2.9 (2.6-3.3)	4.9 (4.5-5.4)	21.3 (20.5-22.1)	30.0 (29.2-30.8)	40.8 (39.9-41.7)	
Straight caregiver	4.6 (3.4-5.7)	8.1 (7.0-9.2)	26.6 (24.9-28.3)	29.1 (27.5-30.8)	31.7 (30.0-33.3)	
Lesbian/Gay non-caregiver	2.6 (0.9-4.3)	10.6 (6.2-14.9)	33.2 (27.5-39.0)	32.1 (26.3-37.9)	21.6 (16.5-26.7)	
Lesbian/Gay caregiver	7.3 (0.2-14.4)	6.5 (2.5-10.5)	38.8 (27.8-49.7)	20.1 (12.5-27.7)	27.3 (16.5-38.2)	
Bisexual non-caregiver	4.2 (2.5-5.9)	12.6 (10.0-15.3)	37.6 (33.6-41.7)	30.2 (26.0-34.4)	15.3 (12.0-18.6)	
Bisexual caregiver	10.8 (4.2-17.5)	15.2 (10.0-20.5)	36.3 (28.1-44.5)	24.8 (17.9-31.6)	12.9 (8.4-17.4)	
"Something else" non-caregiver	6.2 (2.1-10.3)	12.1 (8.6-15.6)	33.2 (27.5-38.9)	26.6 (21.5-31.7)	21.9 (17.0-26.7)	
"Something else" caregiver	14.1 (6.6-21.5)	15.6 (8.6-22.6)	34.7 (24.7-44.7)	25.0 (16.0-34.0)	10.7 (6.0-15.3)	
**Gender Modality and Caregiving Status**						<.001
Cisgender non-caregiver	3.0 (2.6-3.4)	5.4 (5.0-5.9)	22.5 (21.7-23.3)	30.1 (29.3-30.9)	39.0 (38.1-39.9)	
Cisgender caregiver	5.1 (3.9-6.2)	8.5 (7.5-9.5)	27.4 (25.8-29.1)	28.7 (27.1-30.2)	30.3 (28.7-31.8)	
Transgender non-caregiver	9.8 (2.9-16.8)	23.8 (16.0-31.5)	36.7 (28.2-45.3)	20.4 (12.4-28.4)	9.3 (4.5-14.0)	
Transgender caregiver	20.8 (6.4-35.2)	19.1 (6.7-31.5)	35.0 (20.9-49.2)	19.0 (6.8-31.2)	6.0 (1.3-10.8)	

Abbreviation: CI, confidence interval.

© 2024 Xie Z et al. *JAMA Network Open.*

eTable 2. Prevalence of social and emotional support by sexual orientation/gender identity and caregiver status (Question: How often do you get the social and emotional support you need? Is it always, usually, sometimes, rarely, or never?)

	**Prevalence %(95%CI)**					***P***
	Always	Usually	Sometimes	Rarely	Never	
**Sexual Orientation and Caregiving Status**						<.001
Straight non-caregiver	44.8 (43.9-45.7)	32.9 (32.0-33.7)	13.8 (13.1-14.4)	4.5 (4.1-4.9)	4.0 (3.6-4.5)	
Straight caregiver	35.4 (33.6-37.1)	31.9 (30.1-33.6)	20.3 (18.8-21.9)	8.1 (7.0-9.2)	4.3 (3.2-5.4)	
Lesbian/Gay non-caregiver	34.0 (28.0-39.9)	36.7 (31.0-42.4)	21.7 (16.6-26.8)	6.3 (1.6-11.0)	1.3 (0.6-2.1)	
Lesbian/Gay caregiver	32.1 (21.2-43.0)	41.8 (30.4-53.1)	15.2 (9.5-21.0)	10.2 (4.3-16.2)	0.7 (0-1.7)	
Bisexual non-caregiver	22.7 (19.3-26.2)	40.5 (36.3-44.7)	25.5 (21.4-29.7)	7.9 (5.9-10.0)	3.3 (1.7-5.0)	
Bisexual caregiver	23.3 (15.6-30.9)	31.3 (24.1-38.5)	30.3 (22.7-37.8)	9.7 (5.2-14.1)	5.5 (0.7-10.3)	
“Something else” non-caregiver	26.5 (21.0-32.1)	34.4 (28.7-40.1)	23.5 (18.9-28.2)	7.5 (4.4-10.6)	8.0 (4.3-11.7)	
“Something else” caregiver	26.3 (17.2-35.4)	20.2 (13.2-27.1)	33.0 (23.1-42.8)	12.6 (6.3-18.9)	8.0 (1.3-14.6)	
**Gender Modality and Caregiving Status**						<.001
Cisgender non-caregiver	43.3 (42.4-44.2)	33.3 (32.4-34.1)	14.7 (14-15.3)	4.8 (4.3-5.2)	4.0 (3.6-4.4)	
Cisgender caregiver	34.5 (32.9-36.2)	31.8 (30.1-33.4)	20.9 (19.4-22.4)	8.4 (7.4-9.4)	4.4 (3.4-5.5)	
Transgender non-caregiver	19.5 (12.0-27.1)	42.8 (33.7-52.0)	22.1 (15.1-29.2)	9.5 (4.6-14.4)	6.0 (0.1-11.9)	
Transgender caregiver	24.1 (9.8-38.4)	25.6 (12.4-38.9)	38.3 (23.4-53.3)	7.7 (1.3-14.1)	4.2 (0-10.2)	

Abbreviation: CI, confidence interval.

© 2024 Xie Z et al. *JAMA Network Open*.

eTable 3. Participants' caregiving-related characteristics by sexual orientation/gender identity among caregivers **(n=7,765, Weighted n=2,719,664)**

	Caregivers n (weighted %)							
	**Straight**	**Lesbian/ Gay**	**Bisexual**	**Something Else**	***P***	**Cisgender**	**Transgender**	***P***
**Relationship with Care Recipient**					0.14			0.95
Close relatives	5768 (81.7)	118 (77.0)	220 (74.8)	119 (79.2)		6177 (81.2)	48 (80.8)	
Other	1388 (18.3)	43 (23.0)	73 (25.2)	36 (20.8)		1528 (18.8)	12 (19.2)	
**Length of Caregiving**					0.89			0.99
Less than 6 months	2010 (28.1)	53 (29.9)	103 (31.0)	53 (32.3)		2201 (28.4)	18 (27.2)	
6 months to less than 5 years	2929 (40.8)	75 (41.5)	109 (40.5)	56 (34.1)		3145 (40.6)	24 (41.6)	
5 or more years	2217 (31.1)	33 (28.6)	81 (28.5)	46 (33.6)		2359 (30.9)	18 (31.3)	
**Average Hours of Caregiving per Week**					0.62			0.27
Up to 8 hours per week	3968 (53.2)	93 (56.2)	157 (53.5)	95 (60.5)		4285 (53.6)	28 (44.3)	
9 hours or more per week	3188 (46.8)	68 (43.8)	136 (46.5)	60 (39.5)		3420 (46.4)	32 (55.7)	
**Care Recipient's Major Health Problem**					0.35			0.58
Chronic conditions and injury	4036 (56.9)	100 (65.4)	167 (55.7)	92 (63.4)		4360 (57.1)	35 (62.0)	
Old age/others	3120 (43.1)	61 (34.6)	126 (44.3)	63 (36.6)		3345 (42.9)	25 (38.0)	
**Managed Personal Care**					0.45			0.20
Yes	3456 (51.4)	79 (58.2)	148 (56.2)	74 (54.7)		3725 (51.8)	32 (62.2)	
No	3700 (48.6)	82 (41.8)	145 (43.8)	81 (45.3)		3980 (48.2)	28 (37.8)	
**Managed Household Tasks**					0.22			0.79
Yes	5886 (83.7)	145 (91.5)	251 (86.3)	132 (86.3)		6361 (84.0)	53 (85.7)	
No	1270 (16.3)	16 (8.5)	42 (13.7)	23 (13.7)		1344 (16.0)	7 (14.3)	

© 2024 Xie Z et al. *JAMA Network Open*.

eTable 4. Risk of loneliness and lack of social and emotional support in regression analyses among caregivers

	Loneliness	Lack of Social and Emotional Support
	**APR (95% CI)**	**APR (95% CI)**
**Caregiver by Sexual Orientation***		
Straight	Reference	Reference
Lesbian/Gay	1.37 (1.17-1.60)	0.80 (0.57-1.11)
Bisexual	1.25 (1.07-1.47)	1.14 (0.91-1.44)
Something Else	1.18 (0.95-1.46)	1.30 (1.03-1.63)
**Caregiver by Gender Modality****		
Cisgender	Reference	Reference
Transgender	1.41 (1.15-1.74)	1.12 (0.71-1.79)

* adjusted for significant factors including age, sex at birth, marital status, employment status, and number of children in the household
** adjusted for significant factors including age, sex at birth, marital status, household annual income, and number of adults in the household
Abbreviation: APR, adjusted prevalence ratio; CI, confidence interval.

© 2024 Xie Z et al. *JAMA Network Open*.
