# Supplementary material for: Loneliness and Social and Emotional Support Among Sexual and Gender Minority Caregivers
Source: JAMA Netw Open. 2024 Dec 13;7(12):e2451931. doi: 10.1001/jamanetworkopen.2024.51931 (PMC11645647; doi:10.1001/jamanetworkopen.2024.51931)
Supplement: Supplement 2. — Data Sharing Statement [file jamanetwopen-e2451931-s002.pdf]

Data Sharing Statement

Xie. Loneliness and Social and Emotional Support Among Sexual and Gender Minority Caregivers. *JAMA Netw Open*. Published December 18, 2024. doi:10.1001/jamanetworkopen.2024.51931

Data
Data available: No

Additional Information
Explanation for why data not available: The dataset is publicly available on the Centers for Disease Control and Prevention (CDC)'s website.
